# Potency-related effects of smoked cannabis on simulated driving performance: a randomized, controlled crossover trial

**DOI:** 10.1038/s41598-026-43045-2

**Published:** 2026-03-10

**Authors:** Bruna Brands, Adam Zaweel, Madison Wright, Patricia Di Ciano, Christine M. Wickens, Justin Matheson, Andrew Fares, Omer S. M. Hasan, Marcos Sanches, Beth Sproule, Marilyn A. Huestis, Timothy L. Brown, Bernard Le Foll

**Affiliations:** 1 Controlled Substances and Cannabis Branch, Health Canada, Ottawa, ON Canada; 2 Institute for Mental Health Policy Research, Centre for Addiction and Mental Health, 33 Ursula Franklin Street, Toronto, ON M5S 2S1 Canada; 3 Department of Pharmacology and Toxicology, University of Toronto, 27 King’s College Circle, Toronto, ON M5S 3H7 Canada; 4 Translational Addiction Research Laboratory, Centre for Addiction and Mental Health, 33 Ursula Franklin Street, Toronto, ON M5S 2S1 Canada; 5 Dalla Lana School of Public Health, University of Toronto, 155 College Street, Toronto, ON M5T 3M6 Canada; 6 Campbell Family Mental Health Research Institute Centre for Addiction and Mental Health, Ursula Franklin Street, Toronto, ON M5S 2S1 Canada; 7 Institute of Health Policy, Management and Evaluation, University of Toronto, 155 College Street, Suite 425, Toronto, ON M5T 3M6 Canada; 8 Institute of Medical Sciences, University of Toronto, 1 King’s College Circle, Toronto, ON M5S 1A8 Canada; 9 Biostatistician, Biostatistics Core, Centre for Addiction and Mental Health, 60 White Squirrel Way, Toronto, ON M6J 1H4 Canada; 10 Leslie Dan Faculty of Pharmacy, University of Toronto, 144 College Street, Toronto, ON M5S 3M2 Canada; 11 Department of Psychiatry, University of Toronto, 250 College Street, Toronto, ON M5T 1R8 Canada; 12 Pharmacy, Centre for Addiction and Mental Health, 1001 Queen Street, Toronto, ON M6J 1H4 Canada; 13 Institute of Emerging Health Professions, Thomas Jefferson University, 1020 Walnut Street, Philadelphia, PA 19107 USA; 14 Driving Safety Research Institute, University of Iowa, 2401 Oakdale Blvd, Iowa City, IA 52242 USA; 15 Department of Family and Community Medicine, University of Toronto, 500 University Avenue, 5th Floor, Toronto, ON M5G 1V7 Canada; 16 Waypoint Research Institute, Waypoint Centre for Mental Health Care, 500 Church St, Penetanguishene, ON L9M 1G3 Canada

**Keywords:** Cannabis, THC, Simulated driving, Cannabis potency, *Ad libitum*, Drug discovery, Health care, Medical research, Neuroscience

## Abstract

Given the increasing availability of high-potency Δ9-tetrahydrocannabinol (Δ9-THC) products, understanding potency-related effects of Δ9-THC on driving performance is an important public safety issue. This randomized, double-blind, placebo-controlled, within-subjects trial examined the effects of smoked cannabis with varying Δ9-THC concentrations on simulated driving. Adults aged 19–45 who regularly used cannabis and held valid driver’s licenses completed simulated driving tasks after smoking placebo, or cannabis containing low (6.25%/47 mg Δ9-THC), medium (12.5%/94 mg Δ9-THC), or high (22%/165 mg Δ9-THC) Δ9-THC levels. The primary outcome was mean speed (km/h); secondary measures included maximum speed, standard deviation of lateral position (SDLP), reaction time (RT), and subjective ratings of driving ability and intoxication. Mean speed did not differ across conditions. Maximum speed increased under medium (*p* = 0.006) and high (*p* = 0.02) potencies versus placebo. SDLP was higher across all Δ9-THC potencies (*p* < 0.001), and RT was longer under medium and high potencies (*p* < 0.001). Both SDLP (*p* < 0.001) and RT (*p* = 0.023) positively correlated with blood Δ9-THC concentrations. Participants reported poorer driving performance and reduced willingness to drive at higher potencies. Findings demonstrate potency-dependent impairments in simulated driving linked to Δ9-THC concentration, underscoring implications for road safety.

Trial registration: clinicaltrials.gov, ID NCT03656029; First posted date: 04/09/2018; URL: https://clinicaltrials.gov/study/NCT03656029.

**Supplementary Information:**

The online version contains supplementary material available at 10.1038/s41598-026-43045-2.

## Introduction

Delta-9-tetrahydrocannabinol (Δ9-THC) is often detected in drivers who were involved in motor-vehicle collisions^1,2^. According to the Canadian Cannabis Survey, 16% of Canadians who reported past-year cannabis use drove within two hours of smoking or vaping cannabis in 2024^3^. This poses a significant public health and safety concern as meta-analyses showed that driving under the influence of cannabis (DUIC) increases the risk of motor vehicle collisions^4–6^.

Driving simulator studies are an effective method to examine how psychoactive drugs impair driving performance^7^ and showed that cannabis can detrimentally affect measures of lane control (e.g., standard deviation of lateral position, SDLP)^8–13^, reduces speed^11,13–16^, slows reaction time^11,13^, and decreases steering control^15^, though not consistently in all studies. Mixed findings suggest that the impairing effects of cannabis are impacted by other variables, such as Δ9-THC dose and potency of cannabis products^17^. Here, potency typically refers to the percentage of Δ9-THC in a given cannabis product, whereas dose refers to the amount of Δ9-THC administered or consumed. A recent systematic review concluded that the majority of driving studies administered approximately 6% Δ9-THC cannabis that is substantially lower in potency than cannabis products available on the market^18^. In contrast, in North America, the average Δ9-THC potency in dried cannabis significantly increased over the years from 4% in 1995 to about 14% in 2019^19^, with products today containing Δ9-THC as high as 30%^20^. This rise in potency underscores the need to study the effects of escalating Δ9-THC potencies on driving performance.

Prior studies provided evidence of dose- and/or potency-dependent effects of Δ9-THC on SDLP^12,21^, speed^11,13^ and reaction time^11,13^, though typically using a range of lower-potency products. More recently, Marcotte and colleagues^22^ examined driving simulator performance using cannabis potencies of 5.9% and 13.4% Δ9-THC; while there was an effect of cannabis on driving performance (groups receiving active cannabis performed worse in a composite measure of driving simulator performance at 30 min and 90 min after smoking cannabis), there was no difference between the two active conditions. Since 13.4% is still lower compared to what is available on the legal market in Canada and many parts of the US, it is possible that potency-related differences emerge at higher potencies. Our team recently conducted an observational study of driving simulator performance among older adults (aged 65 to 79 years) who smoked higher-potency cannabis (mean 18.74% Δ9-THC); SDLP was increased and mean speed was decreased at 30 min after smoking cannabis compared with the control condition^23^. To address limitations associated with testing low-potency cannabis products that do not reflect the current consumer market, placebo-controlled potency-ranging laboratory studies are needed to examine impairment at higher potencies of Δ9-THC.

The primary aim was to determine the acute effects of varying potencies of smoked cannabis (placebo, low, medium, and high, up to 22% Δ9-THC), on driving simulator performance, hypothesizing that performance would decrease with increasing Δ9-THC potencies. Our primary outcome measure was change in mean speed, given our prior human laboratory study that found significantly decreased mean speed after *ad libitum* smoking of 12.5% Δ9-THC cannabis in young adults^16^. A secondary aim was to examine whether Δ9-THC blood concentrations significantly correlate with driving performance.

## Methods

This study was a within-subject, double-blind, placebo-controlled, randomized and counterbalanced human laboratory study assessing the effects of varying potencies of smoked cannabis (placebo (< 0.1%/0.75 mg Δ9-THC), low-potency (6.25%/47 mg Δ9-THC), medium-potency (12.5%/94 mg Δ9-THC) and high-potency (22%/165 mg Δ9-THC) on simulated driving performance. Cannabis was obtained from Aurora Cannabis Enterprises Inc. Placebo cannabis was provided by the National Institute of Drug Abuse (NIDA) Drug Supply Program. The trial was conducted at a single site in Toronto, Ontario, Canada at the Centre for Addiction and Mental Health (CAMH). Approvals were received from the CAMH (Protocol # 007-2018) and Health Canada (Protocol # 2017-0032) Research Ethics Boards and was registered online on ClinicalTrials.gov (NCT03656029). All study procedures were carried out in accordance with relevant guidelines and regulations, including approvals from both REBs. For detailed methods and additional results not listed here (smoking topography, vital signs, and questionnaires), refer to the Supplementary Materials.

### Participants

Participants were recruited through the community and public transit advertisements in Toronto between June 2021 and June 2022. Participants met inclusion criteria if they were adults aged 19 to 45 years and used cannabis 1–5 days per week but did not meet criteria for cannabis use disorder (to minimize the chance that participants would experience withdrawal the morning of driving assessments). Eligibility also required a positive result for Δ9-THC in either a point-of-care screening or a Clinical Laboratory assay, as confirmed by self-report and urine screening. Additionally, participants had to have held a class G2 or G license, or its equivalent from another jurisdiction, for at least 12 months. They also needed to be willing to abstain from using cannabis for 72 h and from alcohol for 48 h before each practice or test session, and to avoid all other drugs not medically required for the duration of the study, starting 48 h prior to the practice session. Finally, providing written and informed consent was a prerequisite for participation in the study.

Participants were excluded from the study if they had been diagnosed with a severe medical or psychiatric condition, as determined by a qualified investigator (QI), or if they met criteria for current or lifetime alcohol or other substance use disorder according to the Diagnostic and Statistical Manual of Mental Disorders, Fifth Edition (DSM-5), except for tobacco use disorder and caffeine use disorder. Individuals regularly using medication that could affect cognitive functioning and/or driving performance, including antidepressants, benzodiazepines, stimulants, or opioids, were also excluded. Those with a family history of schizophrenia or other psychotic disorders were deemed ineligible. Additionally, exclusion criteria included being pregnant, attempting to become pregnant, or breastfeeding (see Table S1 for details).

### Drug product

Active cannabis was obtained from Aurora, and matching placebo cannabis (< 1% Δ9-THC) was obtained from the National Institute on Drug Abuse (NIDA) in the United States. Each cigarette contained 750 mg of dried plant material with one of four potencies: <1% Δ9-THC (placebo), 6.25% Δ9-THC (low potency), 12.5 ± 2.5% Δ9-THC (medium potency), or 22 ± 4.4% Δ9-THC (high potency). Aurora production of cannabis is highly standardized and secure. Two dried cannabis products were used: one containing 12.5 ± 2.5% Δ9-THC and low cannabidiol (CBD) and a higher-potency product containing 22 ± 4.4% Δ9-THC and < 1% CBD. The 6.25% Δ9-THC condition was generated by mixing 375 mg of the 12.5% product with the placebo cannabis. In March 2022, the 12.5% Δ9-THC strain expired, and the team switched to mixing the 22% Δ9-THC cannabis with placebo to obtain the medium and low potencies.

### Procedure

After pre-screening, participants attended an in-person eligibility assessment where written informed consent was obtained. The study commenced with a practice session, followed by four acute cannabis administration sessions, separated by at least 72 h. Acute administration sessions were identical except for the cannabis potency administered. Participants were randomly assigned to one of four sequences, which were organized in blocks using a Latin square design. Randomization codes were maintained by the CAMH Research Pharmacy.

On testing days, participants completed questionnaires and driving simulation trials, both at baseline and after smoking. Vital signs were monitored, and biological samples (urine, blood, oral fluid) were collected to quantify Δ9-THC concentrations. Blood samples were also analyzed to determine the concentrations of Δ9-THC’s primary metabolites: 11-hydroxy-delta 9-THC (11-OH-Δ9-THC) and 11-nor-9-carboxy-delta 9-tetrahydrocannabinol (Δ9-THC-COOH). Blood samples were collected at baseline, and then 5, 15, 30, 60, 120, 180, 240, 300, and 360 min post-exposure (see supplementary material for full schedule of events). Urine and oral fluid data will be presented elsewhere.

In a dedicated reverse airflow room, participants smoked a single cannabis cigarette containing approximately 750 mg cannabis plant material, with varying Δ9-THC concentrations. To estimate the Δ9-THC “dose” consumed, cannabis cigarettes were weighed before and after smoking (the estimated dose was calculated as change in weight multiplied by the potency of the cannabis product). Cannabis administration initially followed a paced smoking paradigm (53 sessions)^14,24^. During the cued (paced) smoking paradigm, participants followed a PowerPoint presentation, where they were asked to light the cigarette, wait 30 s, inhale for 3 s, hold smoke in their lungs for 7 s, and then exhale. Participants took one puff of the cigarette every minute until the entire cigarette was pyrolyzed. However, this was not well tolerated (e.g., anxiety, paranoia, dizziness, nausea, vomiting, see Table 1) and after consulting with the Drug Safety Monitoring Board, an *ad libitum* smoking procedure was adopted (99 sessions). This procedure, used in our previous studies^16,25^, allowed participants to smoke to their desired effect, with improved tolerability. In the *ad libitum* smoking procedure, participants were instructed to smoke the cigarette as they normally would when smoking cannabis and to only smoke as much as needed to experience the same ‘high’ that they are used to experiencing regularly.

**Table 1:** Adverse events.

Adverse event (AE)	Total	Placebo	Low	Medium	High	Fixed smoking	Ad libitum smoking
Intense cannabis intoxication	5	0	0	0	5	2	3
Nausea/vomiting	5	1	0	0	4	3	2
Fainting	4	2	0	1	1	3	1
Arm soreness, sensitivity, or inflammation due to blood draws	3	0	2	1	0	1	2
Not able to tolerate blood draws	2	0	1	1	0	1	1
Stomachache	1	1	0	0	0	1	0
Headache	1	1	0	0	0	1	0
Cough	1	0	1	0	0	1	0
Tiredness	1	1	0	0	0	1	0
Dizzy	1	1	0	0	0	1	0
Total	24	7	4	3	10	15	9

Total of 24 adverse events reported in 18 participants.

Smoking procedure: 15 AEs occurred in the fixed smoking procedure, and 9 AEs occurred in the *ad libitum* procedure.

Severity: 19 mild, 5 moderate, 0 severe, 0 serious.

Relation to study: 21 probably related, 3 possibly related, 0 unrelated.

After smoking, participants drove the simulator at 30 and 90 min (which reflect the start of the drives) and completed questionnaires related to subjective experience and driving performance (see Table S2 for detailed timeline). All driving measures, aside from reaction time, were assessed under both single- and dual-task driving conditions, with the single-task simulation always preceding the dual-task. The dual-task aimed to increase cognitive load during driving by requiring participants to count backwards in increments of 3 from a number randomly selected between 700 and 999. At the end of the session, participants completed a placebo-effects questionnaire (Table S3), provided biological samples, and were sent home in a taxi. Throughout study visit, participants were monitored for any adverse events (AEs) using a modified version of the SAFTEE questionnaire^26,27^. Based on a list of selected terms, AEs were coded and the date of onset, duration, severity, relationship to study drug, and action taken were recorded.

### Driving simulator

The CAMH Virage VS500M simulator features the driver’s side instrument cluster, steering wheel, controls, and center console of a General Motors compact car. The steering wheel provides dynamic force feedback, as do the brake and accelerator pedals. The visual system consists of three 55-inch screens providing a 180° field of view in the front, and two 17-inch side displays providing visual feedback for the left and right blind zones^16^. Custom driving simulation scenarios are all programmed on the same 9 km 2-lane rural highway. Participants are instructed to maintain a speed of 80 km/h and drive in the center of the lane to the best of their ability, to stay on the main road and drive as they normally would and to interact with other vehicles and obstacles as they would in the real world. Driving assessment includes both a single and a dual task simulation, with the dual task simulation always immediately following the single task simulation. The dual task simulation increases cognitive load and simulates conditions of divided attention. In this dual task simulation, participants count backwards by increments of 3 from a random number between 700 and 999 while driving^28,29^. The addition of a counting backwards task has a long history of use to increase the complexity of cognitive and other tasks^30^. Data was recorded at a frequency of 10 Hz.

A separate driving simulation scenario was programmed to measure reaction time in terms of brake pedal latency. This scenario consists of an endless 4-lane highway where participants were instructed to drive at 100 km/h, while remaining in the second lane to the right. When presented with a true stop sign (stop sign facing them) they were to come to a complete stop as quickly as possible. When presented with a false stop sign (stop sign facing away from them) they were to maintain their speed. During each trial a total of 10 stop signs appeared suddenly at the far-right lane at random intervals, 7 of them were true and 3 of them were false. Data is recorded at a frequency of 60 Hz. The total time spent driving all three driving simulations is approximately 30 min.

### Outcome measures

The primary outcome measure was mean speed (kilometers per hour, km/h), recorded by the driving simulator during pre-programmed driving scenarios. Secondary driving outcomes included maximum speed (MXSP, km/h), standard deviation of speed (SDSP, km/h), standard deviation of lateral position (SDLP, m), and reaction time (RT, s). Other secondary outcome measures included vital signs, oral fluid Δ9-THC concentrations (not presented here), blood Δ9-THC, 11-OH-Δ9-THC and Δ9-THC-COOH concentrations (ng/mL), smoking topography measures, responses from Perceived Driving Ability and Willingness questionnaires, and scores from visual analogue scales (VAS) (Tables S4, S5, S6). (See Supplementary Methods for more details.)

### Data analysis

Linear mixed modelling was used to test our primary aim, with driving outcomes as dependent variables. Condition (Placebo, Low-potency, Medium-potency, High-potency), and condition interacting with time (30 min, 90 min) were the predictors of interest. The models controlled for period (first, second, third, fourth condition), sequence (all possible orders in which the conditions were delivered) and baseline value of the outcome (at time 0 within each condition), with participants included as random effects. Models included completers only, but we also ran additional models following intention to treat (ITT) principles, in that all randomized participants were included in the analysis (including those who withdrew) (see Table S7). To address the secondary aim relating blood Δ9-THC to driving variables, the same model was used and had condition replaced by blood Δ9-THC concentration. These models also controlled for smoking procedure (fixed, *ad libitum*). Mixed-beta regression with logit link modelled VAS after converting the scale from (0,100) to the (0,1) range. Effects were explored using marginal means plots and pairwise group comparisons with p-values non-adjusted for multiple tests, due a smaller-than-planned sample size (see supplementary material). All analyses were conducted in R 4.3.0^31^ using the markdown for reproducibility, packages *lme4*^32^ and *nlme*^33^ for mixed models and *emmeans*^34^ for estimated marginal means.

## Results

### Participants

Of 99 participants screened, 54 were randomized, and 49 received at least one drug condition, though 13 discontinued early (ten due to adverse events, three lost to follow-up) (Fig. S1 for CONSORT diagram). One additional participant was excluded from analysis due to high baseline Δ9-THC concentrations (> 2 ng/ml Δ9-THC), which may indicate protocol non-compliance (i.e., not abstaining from cannabis as instructed). Thus, 35 participants were considered to have completed per-protocol. Participant characteristics are summarized in Table 2. Pairwise comparisons of estimated Δ9-THC dose significantly differed across conditions (*p* < 0.001), increasing with each potency condition (Fig. S2). Smoking topography measures are detailed in Table S6.

**Table 2:** Participant characteristics

Measure	Completers (*n* = 35) Mean (SD) [range] or *n* (%)
*Sex*	
Female	18 (51.4%)
Male	17 (48.6%)
Age (years)	25.23 (3.53) [19–34]
*Race/ethnicity*	
White	16 (45.7%)
West Asian or Arab	5 (14.3%)
Chinese	4 (11.4%)
Filipino	3 (8.6%)
South Asian	2 (5.7%)
Black	2 (5.7%)
Southeast Asian	2 (5.7%)
Korean	1 (2.9%)
Mixed race	0
Latin/Central/South American	0
Height (m)	1.71 (0.10) [1.5–1.9]
Weight (kg)	71.33 (16.4) [40.8–108.9]
BMI (kg/m^2^)	24.10 (4.31) [17.7–36.1]
Weekly cannabis use frequency (days/week)	2.55 (1.2) [1–5]
Amount of Cannabis Used per Using Day (g) in past 3 months	0.68 (0.86) [0.15-5]
Age of Initiation of Cannabis Use (years)	18.31 (3.91) [12–30]
Age of Initiation of Weekly Cannabis Use (years)	21.17 (3.53) [16–30]

### Simulated driving measures

Δ9-THC potency did not impact our primary outcome, mean speed (F(3,203.0) = 0.96, *p* = 0.412). There was a significant main effect of Δ9-THC potency on maximum speed (MXSP) (F(3,212.4) = 2.68, *p* = 0.048); increases in MXSP were significant for the high-potency (Δmean = 2.33 km/h, *p* = 0.02) and medium-potency (Δmean = 2.44 km/h, *p* = 0.01) conditions compared to placebo. There was a main effect of potency on SDSP (F(3,224.6) = 4.03, *p* = 0.008); the high-potency condition had a significantly greater SDSP compared to the medium-potency condition (Δmean = 0.70 km/h, *p* = 0.05) low-potency condition (Δmean = 0.78 km/h, *p* = 0.03) and placebo (Δmean = 1.21 km/h, *p* < 0.001).

**Fig. 1:**
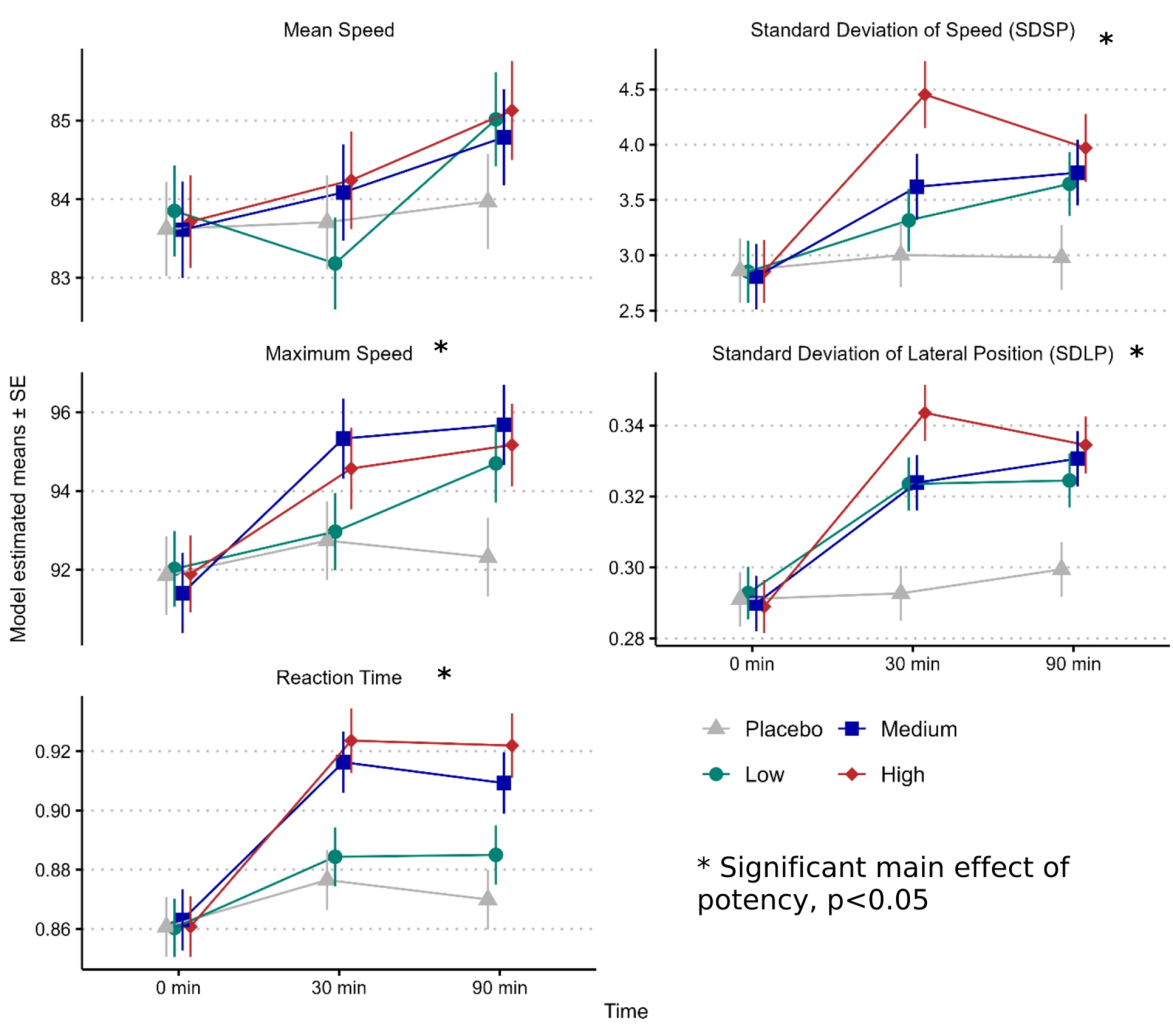
Model estimated means ± standard error of the mean (SEM) for driving performance measures under different Δ9-THC potencies (placebo, low, medium, and high). Mean speed in km/h; SDSP in km/h; maximum speed in km/h; SDLP in m; reaction time in s. Mean speed: no significant effect of potency. SDSP: high > placebo, high > low, high > medium. Maximum speed: high > placebo, medium > placebo. SDLP: high > placebo, medium > placebo, low > placebo, high > low, high > medium. Reaction time: high > placebo, medium > placebo, high > low, medium > low. All pairwise comparisons *p* < 0.05.

There was a main effect of potency on SDLP (F(3,211.6) = 15.8, *p* < 0.001), with all active conditions showing a significant increase in SDLP compared to placebo. The high-potency showed the largest increase relative to placebo (Δmean = 0.041 m, *p* < 0.001), followed by the medium-potency and low-potency, both of which exhibited a similar increase (Δmean = 0.029 m and 0.028 respectively, *p* < 0.001) relative to placebo. The high-potency condition had significantly greater SDLP than both the medium (Δmean = 0.013 m, *p* = 0.04) and low-potencies (Δmean = 0.014 m, *p* = 0.03). There was a significant main effect of potency on RT (F(3,194.4) = 8.0, *p* < 0.001), with RT significantly increased in both the high-potency (Δmean = 0.048s, *p* < 0.001) and medium-potency (Δmean = 0.041s, *p* < 0.001) conditions compared to placebo. Compared to the low-potency condition, RT was significantly elevated in the high-potency (Δmean = 0.037s, *p* < 0.001) and medium-potency (Δmean = 0.030s, *p* < 0.001). See Fig. 1. Of note, there were no significant time by potency interactions, suggesting that the potency effect was similar at 30 and 90 min.

Results from the dual-task conditions were similar to results in the single-task and are presented in Table S7, which also presents a comparison of the ITT (all participants) and PP (completers only) analysis, which did not show meaningful differences. A summary of pairwise comparisons can be found in Table S8.

### Driving ability

**Fig. 2:**
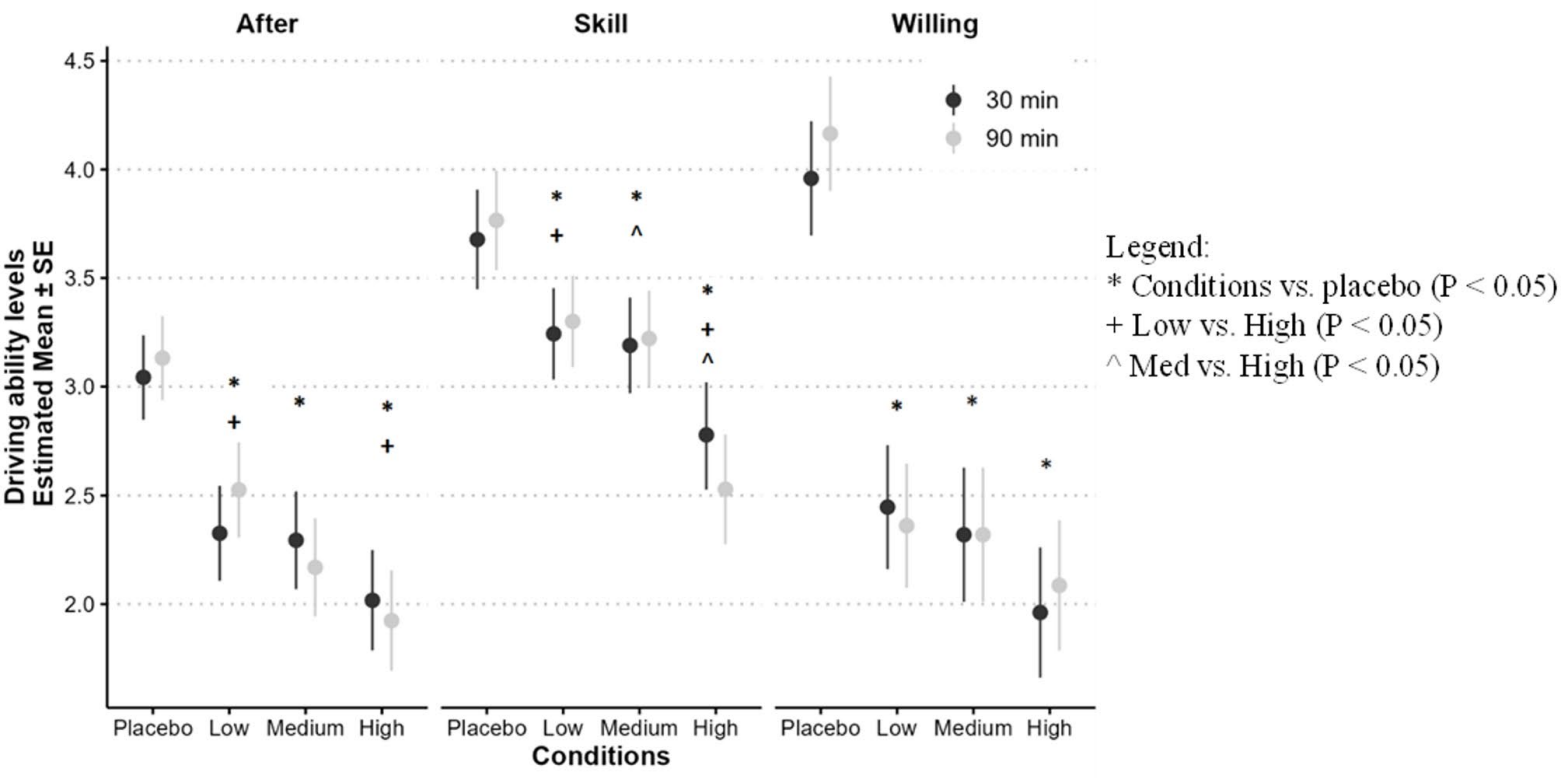
Self-perceived driving ability at 30 and 90 min under different Δ9-THC potencies (placebo, low, medium, and high). Data are presented as model estimated means ± SEM. All scales range from 0 to 5.

We assessed participants’ subjective evaluation of their driving skills (‘Skill’), willingness to drive in their current state (‘Willing’), and perceived driving ability relative to their regular, non-intoxicated state (‘After’). Significant main effects were observed across all measures: ‘Skill’ (F(3,31.4) = 5.88, *p* = 0.003), ‘Willing’ (F(3,32.2) = 21.39, *p* < 0.001), and ‘After’ (F(3,31.50) = 12.15, *p* < 0.001). Participants rated their ‘Skill’ lower in the high-potency condition compared to the medium-potency (Δmean=-0.55, *p* = 0.014), low-potency (Δmean=-0.62, *p* = 0.005), and placebo conditions (Δmean=-1.07, *p* < 0.001). Ratings for ‘Willing’ and ‘After’ exhibited similar patterns, with higher scores in the placebo condition than the active conditions (Fig. 2).

### Smoking topography

There was a significant main effect of potency on amount smoked (*p* < 0.001), smoking duration (*p* = 0.005), and estimated Δ9-THC dose (*p* < 0.001), but not number of puffs (*p* = 0.052) (Fig. S2). Participants generally smoked less and for shorter periods as potency increased. Specifically, participants smoked significantly less in the high-potency condition compared to all other conditions (*p* < 0.001) and for a shorter duration in the high-potency condition compared to the medium-potency condition (Δmean=-0.70 *p* = 0.042) and the low-potency condition (Δmean=-1.17, *p* = 0.001). Finally, all pairwise comparisons significantly differed for estimated Δ9-THC dose, which increased with increasing potency condition (*p* < 0.001). Summarized in Table S6.

### Subjective effects (VAS)

VAS ratings were significantly higher in the active potency conditions compared to placebo (*p* < 0.001), with subjective intoxication observed at the time of driving stimulator assessments (30 and 90-minutes). The high potency showed a general pattern of higher VAS scores compared to medium and low potencies (Fig. S3).

### Δ9-THC concentrations in whole blood

There was a significant main effect of condition for Δ9-THC (F(3,82.35) = 11.4, *p* < 0.001), 11-OH-Δ9-THC (F(3,34.45) = 11.4, *p* < 0.001), and Δ9-THC-COOH (F(3,29.57) = 15.1, *p* < 0.001) (Fig. S4). Smoking procedure (i.e., fixed vs. *ad libitum* smoking) did not meaningfully impact blood Δ9-THC or metabolite concentrations, as there was no main effect of smoking procedures on blood Δ9-THC (F(1,170.37) = 0.36, *p* = 0.55), 11-OH-Δ9-THC (F(1,619.62) = 0.14, *p* = 0.71), or Δ9-THC-COOH (F(1,31.38) = 3.6, *p* = 0.068) concentrations. See Table 3 for descriptive means of Δ9-THC.

**Table 3:** Blood cannabinoid concentrations (descriptive means; in ng/mL).

Time (min)	Placebo		Low		Medium		High	
	Mean	SD	Mean	SD	Mean	SD	Mean	SD
0	0.23	0.52	0.14	0.31	0.16	0.41	0.22	0.51
5	0.20	0.48	16.06	15.30	19.71	24.52	30.32	35.13
15	0.23	0.54	6.45	5.93	8.11	8.91	14.89	13.81
30	0.18	0.43	4.81	4.63	5.65	5.82	9.05	7.67
60	0.20	0.49	2.89	2.66	3.81	3.97	6.07	5.36
90	0.20	0.52	2.34	2.20	2.59	2.55	4.00	3.75
120	0.19	0.52	1.54	1.61	1.60	1.81	2.58	2.55
180	0.20	0.49	0.86	1.26	0.94	1.16	1.16	1.34
240	0.23	0.56	0.61	1.00	0.56	0.98	0.72	0.89
300	0.29	0.60	0.46	0.83	0.46	0.83	0.52	0.75
360	0.26	0.64	0.37	0.70	0.38	0.75	0.35	0.71

### Δ9-THC concentrations in biological fluid and driving outcomes

**Fig. 3:**
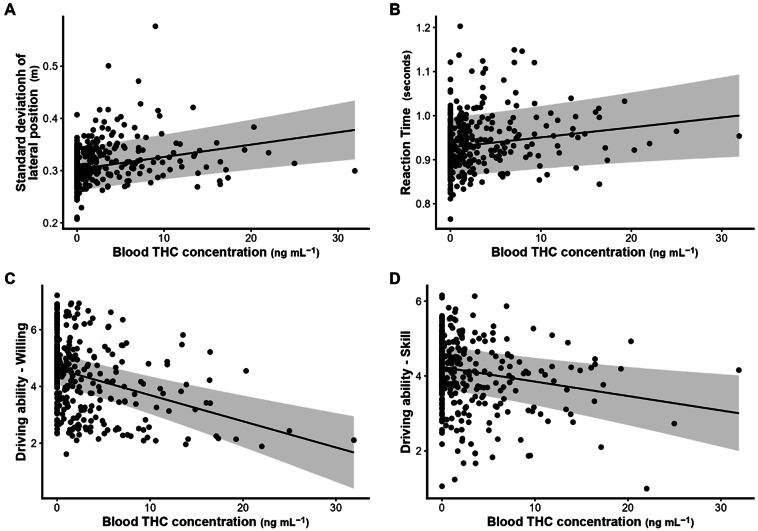
Partial residual plots of driving performance metrics in relation to blood Δ9-THC concentrations: (**A**) SDLP, (**B**) reaction time, (**C**) willing, and (**D**) skill.

Blood Δ9-THC was significantly positively correlated with SDLP (slope = 0.0024, SE = 0.0006, *p* < 0.001) and reaction time (slope = 0.002, SE = 0.0009, *p* = 0.023). Blood Δ9-THC was significant negatively correlated with driving ability questionnaires, including “Skill” (slope = – 0.038, *p* = 0.004), “Willing” (slope = – 0.092, *p* < 0.001), and “After” (slope = – 0.086, *p* < 0.001), indicating decreased perceived driving ability and willingness with higher Δ9-THC concentrations (Fig. 3). Similar correlations across all driving outcome measures were observed for blood 11-OH-Δ9-THC and Δ9-THC-COOH concentrations (Table S9). We ran additional models controlling for Δ9-THC condition; while some correlations were no longer significant when condition when entered into the models, many of the significant correlations remained (Table S9).

### Adverse events

A total of 24 adverse events (AEs) were reported among 18 (out of 49) participants, with 19 classified as mild and 5 as moderate in severity (Table 1). Of the 24 AEs reported, 15 occurred during the fixed smoking procedure and 9 in the *ad libitum* smoking procedure. More incidents of fainting and nausea/vomiting were observed in the fixed smoking procedure compared to the *ad libitum* paradigm.

## Discussion

In this randomized, placebo-controlled crossover trial, smoked cannabis negatively impacted driving simulator performance. Medium (12%) and high (22%) Δ9-THC potencies consistently increased maximum speed, variability of speed, SDLP, and reaction time. However, our primary outcome (mean speed), selected based on our previous study^16^, was unaffected. These objective measures of impairment were paralleled by subjective feelings of intoxication and self-reported declines in driving ability and willingness to drive.

Evidence for potency-related effects was mixed. Significant differences in performance measures (SDSP, SDLP, and subjective driving assessments) were observed between the high-potency condition and the medium- and low-potency conditions, as well as between all active-potency conditions and placebo. Differences between medium and low potencies were minimal, consistent with a recent study testing doses similar to our medium and low doses^22^, suggesting comparable impairments within the 6–13% Δ9-THC range. Our findings suggest that driving impairments increase with higher Δ9-THC potencies (around 22%), beyond those previously studied. Furthermore, our results suggested that differences in driving performance were similar at 30 and 90 min after smoking cannabis. Our findings are important for traffic safety, as higher SDLP can lead to more lane crossings (e.g., into oncoming traffic, or into the road shoulder), and is considered an index of safe driving^35^. The difference in SDLP between the high-potency and placebo conditions in our study (4.3 cm) exceeded the difference from placebo at a blood alcohol concentration (BAC) of 0.05% in a previous study (2.4 cm) and was identical to the difference at 0.08% (4.3 cm)^35^. Thus, our results suggest that there is a clinically significant level of SDLP increase at our high-potency condition relative to placebo.

It is important to note that while we administered a higher-potency cannabis product than in previous studies, our blood Δ9-THC concentrations were not necessarily higher than in previous studies. For example, Marcotte and colleagues^22^ observed mean Δ9-THC concentrations of 50.6 and 29.3 ng/mL 15 min after smoking 5.9% and 13.4% Δ9-THC cannabis, respectively. These concentrations were considerably higher than the equivalent mean Δ9-THC concentrations we saw at 15 min post-smoking in our 12.5% condition (8.11 ng/mL) and 22% condition (14.89 ng/mL). Further work is clearly needed to parse apart relationships between cannabis potency, blood Δ9-THC concentrations, and performance outcomes.

Significant correlations were observed between Δ9-THC concentrations in blood and both objective and subjective measures of driver impairment, which was more consistently evident when not controlling for Δ9-THC, though many correlations remained even when Δ9-THC condition was entered into the models. This relationship has been difficult to establish^22,36–38^, likely due to Δ9-THC’s complex pharmacology. For example, Δ9-THC can persist in brain tissue even when undetectable in blood^39^, which may explain poor correlation between peripheral blood Δ9-THC concentrations and acute effects^40^, a long-recognized challenge for roadside enforcement of cannabis-involved driving^41^. A linear dose-response may not be evident at lower potencies, but could emerge at higher concentrations, as seen in our data and supported by recent epidemiological reviews^42,43^. However, further work is needed to substantiate this hypothesis.

Our study is one of the first to test potency-response effects of cannabis on driving abilities incorporating such a high potency. We transitioned from a fixed smoking paradigm, aimed at standardizing Δ9-THC exposure across different conditions, to an *ad libitum* approach due to the poor tolerability of the fixed-smoking paradigm at higher potencies. The high incidence of adverse events, despite including participants who regularly used cannabis, illustrates the potential risk associated with cannabis administration studies administering higher-potency products. It is interesting to note that, while participants generally smoked less as potency increased (suggesting some degree of self-titration), estimated Δ9-THC dose still significantly increased with increasing potency. We will explore this further in a future manuscript.

The study’s strengths include the robust placebo-controlled, crossover design and the wide range of cannabis potencies administered. Limitations include the use of two paradigms for administering cannabis, which may have influenced the results by over or under-estimating Δ9-THC dose. Note, however, that fixed smoking paradigms do not completely standardize dose. For example, one study that used a fixed cannabis smoking paradigm found a mean observed peak serum Δ9-THC of 120.9 ng/mL in adults reporting heavy use of cannabis compared to just 49.1 ng/mL in a group of adults reporting occasional use^44^. Furthermore, we controlled for smoking procedure in all relevant statistical models, and we also showed no main effect of smoking procedure on blood Δ9-THC or metabolite concentrations. There was a change in our procedure for generating the low and medium cannabis cigarettes towards the end of the study; as we did not verify the Δ9-THC content of each cannabis cigarette, it is possible there could be variability in the amount of Δ9-THC between participants. We chose to recruit a sample of participants endorsing regular cannabis use, which is in line with our goal to test higher-potency cannabis products. Our findings are unlikely to generalize beyond this participant sample (e.g., people using very infrequently or people with a cannabis use disorder may have different responses to cannabis). Additionally, blood samples were analyzed by two different laboratories with slightly different analytical method (GCMS vs. LCMS/MS, see supplementary material). Although both methods are highly comparable, differences in methodology are noted as a limitation. Finally, driving simulator trials were conducted at 30 and 90 min post-exposure; thus, we cannot determine the impact on driving abilities beyond 90 min or when they return to baseline.

In conclusion, smoked cannabis significantly impaired simulated driving performance and self-perceived driving ability, especially at 22% Δ9-THC, and these impairments were correlated with blood Δ9-THC concentrations. While questions remain about impairment at low Δ9-THC potencies and impairment duration, our findings support public health efforts to warn against driving after smoking high-potency cannabis, including public education campaigns highlighting the risks associated with potent cannabis products. Clinicians, well-positioned to counsel patients, should ask about cannabis use and potency to stress the increased driving risks posed by high-Δ9-THC products. As high-potency cannabis becomes more prevalent, expanding research is essential to inform public health strategies.

## Supplementary Information

Below is the link to the electronic supplementary material.

Supplementary Material 1

Supplementary Material 2

Supplementary Material 3

Supplementary Material 4

Supplementary Material 5

## Data Availability

The datasets used and/or analysed during the current study available from the corresponding author on reasonable request.
