# Supplementary material for: Potency-related effects of smoked cannabis on simulated driving performance: a randomized, controlled crossover trial
Source: Sci Rep. 2026 Mar 10;16:12961. doi: 10.1038/s41598-026-43045-2 (PMC13096524; doi:10.1038/s41598-026-43045-2)
Supplement: Supplementary file 1 — Supplementary Material 1 [file 41598_2026_43045_MOESM1_ESM.docx]

**Potency-Related Effects of Smoked Cannabis on Simulated Driving Performance: A Randomized, Controlled Crossover Trial**

**Supplemental Appendix**

**S1. Supplementary Methods**1

**S2. Supplementary Results**2

**TABLES & FIGURES**

**Table S1.** Inclusion and Exclusion Criteria. 4

**Table S2.** Schedule of drug administration and data collection.4

**Table S3.** Placebo Questionnaire 5

**Table S4.** Driving Ability and Willingness Questionnaire5

**Table S5.** Visual Analogue Scales 6

**Figure S1.** CONSORT Diagram.6

**Figure S2.** Cannabis Smoking Topography7

**Table S6.** Marginal Means for Smoking Topography Measures……………………………………7

**Table S7.** ANOVA Results for the Effect of Δ9-THC potency on Driving Performance Outcomes 8

**Table S8.** Pairwise Comparisons of Δ9-THC potency Effects on Driving Performance Measures 9

**Figure S3.** VAS11

**Table S9.** Estimated Slopes for the blood Δ9-THC correlations12

**Figure S4.** Estimated mean Δ9-THC and metabolites over time12

**Figure S5.** Vital Signs13

**S1. Supplementary Methods:**

**Confirmation of ongoing eligibility**

Practice and test sessions began with confirmation of participants’ continued eligibility. This included Breath Alcohol Content (BrAC) testing using the AlertTM J5 model Breathalyzer (Alcohol Countermeasure Systems) to confirm a BrAC level of 0, urine toxicology screenings via the QuickscreenTM CLIA-Waived 10-Panel Multi Drug Test, cannabis saliva tests with Securetec DrugWipe^®^ 3s at 25 ng/mL, and self-reports via the Cannabis Timeline Follow-Back^1^ to confirm compliance with the abstention period required for alcohol, cannabis, and all other drugs not medically indicated. Female participants were also required to test negative on a urine pregnancy test at each session.

**Test Sessions**

All drug administration sessions were identical except for the drug condition. Following confirmation of ongoing eligibility, participants completed baseline subjective effects and driving ability questionnaires, followed by a practice driving trial and baseline driving trials. Baseline urine, blood, and oral fluid samples were also collected for Δ9-THC and metabolites (11-OH-Δ9-THC, and Δ9-THC-COOH) quantification, as well as vital signs. At baseline, a registered nurse inserted an in-dwelling intravenous catheter in the forearm to facilitate the collection of serial blood draws over 6 hours.

**Cannabinoid Quantification**

Due to unforeseen staffing shortages at the CAMH Laboratory, blood samples were analyzed by Dynacare Medical Laboratories and the CAMH Clinical Laboratory. During the transfer from CAMH to Dynacare, samples were frozen at different temperatures for various periods of time, initially they were stored in -20C, moved to -80C, and then moved again to -30C at Dynacare. The CAMH Laboratory analyzed the samples using Gas Chromatography-Mass Spectrometry (GCMS) while Dynacare Medical Laboratories used Liquid Chromatography-Tandem Mass Spectrometry (LCMS/MS).

**Blood Cannabinoid Levels:** Δ9-THC and its two main metabolites, 11-hydroxy-delta-9-tetrahydrocannabinol (11-OH-Δ9-THC), and 11-nor-9-carboxy-delta-9-tetrahydrocannabinol (Δ9-THC-COOH), were measured in whole-blood samples taken at specified intervals - immediately after, and 5, 15, and 30 minutes, as well as 60, 90, 120, 180, 240, 300, and 360 minutes post-cannabis administration. A registered nurse collected each 10 mL sample using a Becton-Dickinson Vacutainer® containing K2 EDTA, which was then stored in a Simport Cryovial T310-10A Polypropylene Vial and frozen at temperatures of -20°C, then -80°C, and finally -30°C. The CAMH Clinical Laboratory analyzed the first 10 participants' samples, and Dynacare analyzed the remaining 26 due to unforeseen staffing shortages. The CAMH lab used gas chromatography-mass spectrometry, and Dynacare used liquid chromatography-tandem mass spectrometry for analysis. The quantification limits for Δ9-THC, 11-OH-Δ9-THC, and Δ9-THC-COOH were set at 0.5, 1.0, and 1.0 ng/ml, respectively, with values below these thresholds recorded as zero.

**Sample Size Calculation and Interim Analysis**

The initial target sample size was 50 subjects, based on a power calculation conducted using data from a similar study from our lab.^2^ However, due to a looming expiry of the cannabis supply, and regulatory challenges with obtaining a new supply of cannabis, only 36 subjects were recruited for this study. Hence, we used the collected data and our primary aim to estimate the detectable effect size given our achieved sample size, 80% power, and a mixed model that targets condition by time interaction as effect of interest, while controlling for period, sequence and baseline mean speed. Simulations were conducted in R using package simr^3^ and we found that this sample is sufficient to detect an effect equivalent to partial eta-squared 0.06 (small effect = 0.01, medium effect = 0.06, large effect = 0.11).

When 18 participants had completed the study, an interim analysis was conducted for a student's PhD thesis. To maintain study integrity, the student was recused from daily operations and was the only team member with access to the unblinded data. The study was not planned to stop based on interim results and continued until the target sample size was reached.

**S2. Supplementary Results:**

**Simulated Driving Measures (Dual-task Conditions)**

In dual-task driving simulations, we found no significant change in mean speed (F=0.8, p=0.48) or maximum speed (MXSP) (F=2.3, p=0.08) across different Δ9-THC potencies. There was a main effect of potency on the SDSP (F=34.0, p=0.009), with the high-potency condition (Δmean=1.16 km/h, p<0.001) and medium-potency condition (Δmean=1.09 km/h, p<0.001) showing a significant increase in variability compared to placebo. Similar to single-task conditions, a significant main effect was found for the standard deviation of lateral position (SDLP) under dual-task conditions (F=2.8, p=0.042), with the high-potency (Δmean=0.025m, p=0.02) and medium-potency (Δmean=0.032m, p=0.01) showing increased SDLP compared to placebo. (**Table S7 and S8**)

**Vital Signs (Figure S5)**

We found no evidence of a potency effect for Systolic Blood Pressure (p = 0.112) (Figure S5). There was strong evidence for a time by potency interaction effect of Heart Rate (p<0.001, Figure S5). This interaction effect was characterized by increased heart rate being associated with increased potency at the initial times after exposure to cannabis and converging to similar heart rates at later times.

**Placebo Questionnaire:**

Participants were asked to rate the strength of the cannabis they received relative to their usual cannabis. Most (91.4%) participants identified the placebo as "much weaker", while perceptions of active cannabis strength varied: 11.4%, 20%, and 9.1% respectively categorized the low, medium, and high-potencies as "much weaker." In the high-potency condition, 36.36% and 9.09%, perceived the cannabis as "somewhat stronger" and "much stronger," respectively. Participants were also asked to identify whether they received placebo or active cannabis (regardless of potency). A large percentage of participants correctly identified their condition across all levels: 82.9%, 94.3%, 91.4%, and 90.9% for the placebo, low, medium, and high conditions respectively (**Table S3**).

| **Table S1:** Inclusion and Exclusion Criteria. | |
| --- | --- |
| **Inclusion:** | **Exclusion:** |
| Males and females aged 19 to 45 years | Diagnosis of severe medical or psychiatric condition (as judged by Qualified Investigator) |
| Near-daily use of cannabis (1-5 days/week) confirmed by self-report and urine screening (i.e., positive cannabinoid result in point-of-care screening or Clinical Laboratory assay) | Meets criteria for current or lifetime alcohol or other substance use disorder (DSM-5), except tobacco use disorder and caffeine use disorder |
| Has held a class G2 or G license (or equivalent from another jurisdiction) for at least 12 months | Regular use of medication that may affect cognitive functioning and/or driver performance (e.g. antidepressants, benzodiazepines, stimulants, opioids) |
| Willing to abstain from using cannabis for 72 hours prior to each practice or test session | Family history of schizophrenia or other psychotic disorder |
| Willing to abstain from alcohol for 48 hours prior to each practice or test session, and to abstain from all other drugs not medically required for the duration of the study (beginning 48 hours prior to the practice session) | Pregnant, looking to become pregnant, or breastfeeding |
| Provides written and informed consent |  |

| **Table S2:** Schedule of drug administration and data collection. | | | | | | | | | | | | | |
| --- | --- | --- | --- | --- | --- | --- | --- | --- | --- | --- | --- | --- | --- |
| ` | **Eligibility** | **Practice** | **Drug Administration Sessions (1-4)**  **End of smoking marks Time 0**  **↓** | | | | | | | | | | |
|  |  |  | Baseline  -120m | 5  m | 15m | 30m | 60m | 90m | 2h | 3h | 4h | 5h | 6h |
| **Driving Trial** |  | **X** | **X** |  |  | **X** |  | **X** |  |  |  |  |  |
| Breath tests (alcohol) | **X** | **X** | **X** |  |  |  |  |  |  |  |  |  |  |
| Timeline follow back for cannabis use | **X** | **X** | **X** |  |  |  |  |  |  |  |  |  |  |
| Physical Exam, Psychiatric Exam (SCID) | **X** |  |  |  |  |  |  |  |  |  |  |  |  |
| Vital Signs | **X** |  | **X** | **X** | **X** | **X** | **X** | **X** | **X** | **X** | **X** | **X** | **X** |
| Urine: Point-of-care drug screen | **X** | **X** | **X** |  |  |  |  |  |  |  |  |  |  |
| Urine: quantitative Δ9-THC and Δ9-THC metabolites normalized to creatinine | **x** | **x** | **X** |  |  |  |  |  |  |  |  |  | **X** |
| Urine: Point-of-care pregnancy test | **X** | **X** | **X** |  |  |  |  |  |  |  |  |  |  |
| Blood: Biochemistry, Hematology | **X** |  |  |  |  |  |  |  |  |  |  |  |  |
| Blood: Δ9-THC and metabolites quantification |  |  | **X** | **X** | **X** | **X** | **X** | **X** | **X** | **X** | **X** | **X** | **X** |
| Oral fluid: Δ9-THC and metabolites quantification |  |  | **X** |  |  | **X** |  | **X** | **X** |  |  |  | **X** |
| Saliva: Δ9-THC detection | **X** | **X** | **X** |  |  | **X** |  | **X** | **X** |  |  |  | **X** |
| VAS |  | **X** | **X** | **X** | **X** | **X** | **X** | **X** | **X** | **X** | **X** | **X** | **X** |
| Driving ability questionnaires |  |  | **X** |  |  | **X** |  | **X** |  |  |  |  |  |
| Placebo questions |  |  |  |  |  |  |  |  |  |  |  |  | **X** |

| **Table S3.1:** Strength Perception  **Question:** Please rate the strength of the cannabis you received today in relation to the cannabis that you usually smoke. Would you say it is much weaker than your usual cannabis? Much weaker? Somewhat weaker? About the same? Somewhat stronger? Or much stronger? | | | | |
| --- | --- | --- | --- | --- |
| **Response** | **Condition** | | | |
|  | **Placebo (%)** | **Low (%)** | **Medium (%)** | **High (%)** |
| Much Weaker | 91.4 | 11.4 | 20.0 | 9.1 |
| Somewhat Weaker | 5.7 | 34.3 | 28.6 | 12.1 |
| About the Same | 2.9 | 31.4 | 28.6 | 33.3 |
| Somewhat Stronger | 0.0 | 17.1 | 22.9 | 36.4 |
| Much Stronger | 0.0 | 5.7 | 0.0 | 9.1 |

**Table S3.** Placebo Questionnaire.

| **Table S3.2:** Cannabis Type  **Question:** If you had to guess, would you say you received the placebo cannabis, or the active cannabis? (Specify) | | | | |
| --- | --- | --- | --- | --- |
| **Response** | **Condition** | | | |
|  | **Placebo (%)** | **Low (%)** | **Medium (%)** | **High (%)** |
| **Guessed Correctly** | 82.9 | 94.3 | 91.4 | 90.9 |

| **Table S4:** Driving Ability and Willingness Questionnaire | | | | | |
| --- | --- | --- | --- | --- | --- |
| Please rate your demonstrated skill during the simulation | 1 -  I demonstrated POOR driving skills | 2 | 3 | 4 | 5 -  I demonstrated EXCELLENT driving skills |
| For a moment, think about how you were feeling physically, mentally, and emotionally before you began the driving simulation. If you were feeling the same way now, how willing would you be to drive a real vehicle? | 1 -  Not at all willing to drive a real vehicle | 2 | 3 | 4 | 5 -  Very willing to drive a real vehicle |
| Using the scale below, how do you think your driving skills during the simulation compared to how you might have driven without smoking the cigarette beforehand? | 1 -  My driving skills were WORSE THAN if I had not smoked the cigarette | 2 | 3 -  My driving skills were THE SAME AS if I had not smoked the cigarette | 4 | 5 -  My driving skills were BETTER THAN if I had not smoked the cigarette |

**Table S5:** Visual Analogue Scales

| I like this drug effect: | 0 50 100  -------------------□------------------- |
| --- | --- |
| This feels like cannabis: | 0 50 100  -------------------□------------------- |
| I feel this effect: | 0 50 100  -------------------□------------------- |
| I feel this high: | 0 50 100  -------------------□------------------- |
| I feel the good effects: | 0 50 100  -------------------□------------------- |
| I feel the bad effects: | 0 50 100  -------------------□------------------- |
| I feel the rush: | 0 50 100  -------------------□------------------- |

**Figure S1** CONSORT Diagram outlining participant screening, enrollment, randomization, and exclusions.

**Figure S2.** Smoking topography measures by cannabis condition. Estimated Δ9-THC dose in mg; Puffs in number of puffs during the smoking session; Time in min; Weight is change of the cannabis cigarette weight from before to after smoking in mg

| **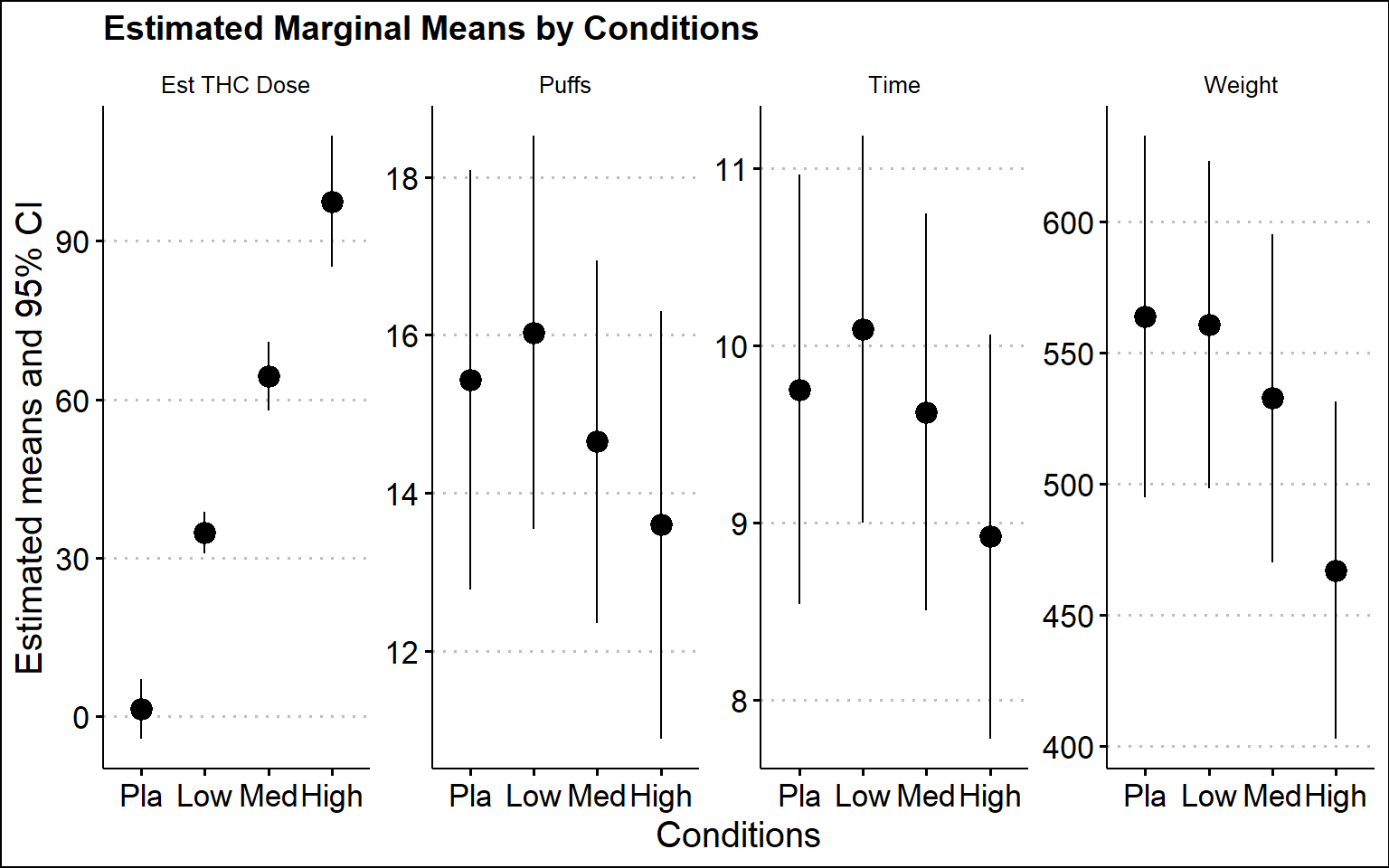**  **Table S6.** Estimated Marginal Mean Differences for Various Smoking Topography Outcomes | | | | | | | |
| --- | --- | --- | --- | --- | --- | --- | --- |
| **Outcome Measure** | **Contrast** | **Estimate (Δmean)** | **SE** | **t.ratio** | **p.value** | **Cohens_d** | **Cohen’s d CI (Lower, Upper)** |
| Amount Smoked (mg) | Pla - Low | 3.001 | 21.079 | 0.142 | 0.887 | 0.030 | (-0.377, 0.436) |
| Amount Smoked (mg) | Pla - Med | 31.047 | 20.147 | 1.541 | 0.127 | 0.320 | (-0.090, 0.728) |
| Amount Smoked (mg) | Pla - High | 96.677 | 21.407 | 4.516 | **<0.001*** | 0.937 | (0.510, 1.362) |
| Amount Smoked (mg) | Low - Med | 28.045 | 14.469 | 1.938 | 0.056 | 0.402 | (-0.010, 0.811) |
| Amount Smoked (mg) | Low - High | 93.676 | 16.154 | 5.799 | **<0.001*** | 1.203 | (0.758, 1.641) |
| Amount Smoked (mg) | Med - High | 65.631 | 14.581 | 4.501 | **<0.001*** | 0.934 | (0.503, 1.359) |
| Number of Puffs (n) | Pla - Low | -0.599 | 0.988 | -0.606 | 0.546 | -0.126 | (-0.532, 0.282) |
| Number of Puffs (n) | Pla - Med | 0.781 | 0.819 | 0.953 | 0.343 | 0.198 | (-0.210, 0.605) |
| Number of Puffs (n) | Pla - High | 1.836 | 1.084 | 1.693 | 0.094 | 0.351 | (-0.059, 0.760) |
| Number of Puffs (n) | Low - Med | 1.380 | 0.697 | 1.979 | 0.051 | 0.410 | (-0.001, 0.820) |
| Number of Puffs (n) | Low - High | 2.435 | 0.994 | 2.448 | **0.016*** | 0.508 | (0.093, 0.919) |
| Number of Puffs (n) | Med - High | 1.055 | 0.816 | 1.292 | 0.200 | 0.268 | (-0.141, 0.676) |
| Smoking Duration (minutes) | Pla - Low | -0.341 | 0.413 | -0.826 | 0.411 | -0.171 | (-0.578, 0.236) |
| Smoking Duration (minutes) | Pla - Med | 0.128 | 0.412 | 0.310 | 0.758 | 0.064 | (-0.343, 0.471) |
| Smoking Duration (minutes) | Pla - High | 0.829 | 0.428 | 1.934 | 0.056 | 0.401 | (-0.011, 0.811) |
| Smoking Duration (minutes) | Low - Med | 0.469 | 0.326 | 1.439 | 0.153 | 0.299 | (-0.111, 0.706) |
| Smoking Duration (minutes) | Low - High | 1.170 | 0.345 | 3.388 | **0.001*** | 0.703 | (0.282, 1.120) |
| Smoking Duration (minutes) | Med - High | 0.701 | 0.340 | 2.064 | **0.042*** | 0.428 | (0.016, 0.838) |
| Estimated Δ9-THC Dose (mg) | Pla - Low | -33.336 | 1.986 | -16.783 | **<0.001*** | -3.481 | (-4.121, -2.833) |
| Estimated Δ9-THC Dose (mg) | Pla - Med | -62.886 | 3.169 | -19.844 | **<0.001*** | -4.116 | (-4.829, -3.395) |
| Estimated Δ9-THC Dose (mg) | Pla - High | -96.014 | 6.058 | -15.849 | **<0.001*** | -3.287 | (-3.906, -2.660) |
| Estimated Δ9-THC Dose (mg) | Low - Med | -29.550 | 2.475 | -11.940 | **<0.001*** | -2.476 | (-3.013, -1.932) |
| Estimated Δ9-THC Dose (mg) | Low - High | -62.679 | 5.724 | -10.951 | **<0.001*** | -2.271 | (-2.788, -1.746) |
| Estimated Δ9-THC Dose (mg) | Med - High | -33.129 | 6.228 | -5.319 | **<0.001*** | -1.103 | (-1.537, -0.664) |
| Pairwise comparisons of smoking topography outcomes across different conditions (*p < 0.05 indicates significant differences) CI: Confidence Interval | | | | | | | |

| **Table S7.** ANOVA results for the effect of Δ9-THC potency on driving performance outcomes (two models shown: ITT and PP) | | | | | | | | | | | |
| --- | --- | --- | --- | --- | --- | --- | --- | --- | --- | --- | --- |
| **Driving Outcome / Driving Attention** | | | | **NumDF** | | **DenDF** | | **F value** | | **Pr(>F)** | |
| MSP - Single Task (ITT) | | | | 3 | | 227.715 | | 0.962 | | 0.412 | |
| MSP - Dual Task (ITT) | | | | 3 | | 225.876 | | 1.038 | | 0.376 | |
| MXSP - Single Task (ITT) | | | | 3 | | 229.614 | | 2.835 | | **0.039*** | |
| MXSP - Dual Task (ITT) | | | | 3 | | 227.889 | | 2.665 | | **0.049*** | |
| SDLP - Single Task (ITT) | | | | 3 | | 230.826 | | 15.899 | | **<0.001*** | |
| SDLP - Dual Task (ITT) | | | | 3 | | 221.647 | | 3.360 | | **0.019*** | |
| SDSP - Single Task (ITT) | | | | 3 | | 249.222 | | 4.343 | | **0.005*** | |
| SDSP - Dual Task (ITT) | | | | 3 | | 239.891 | | 4.029 | | **0.008*** | |
| Reaction Time (ITT) | | | | 3 | | 222.027 | | 8.540 | | **<0.001*** | |
| MSP - Single Task (PP) | | | | 3 | | 203.005 | | 0.960 | | 0.412 | |
| MSP - Dual Task (PP) | | | | 3 | | 213.019 | | 0.823 | | 0.483 | |
| MXSP - Single Task (PP) | | | | 3 | | 212.428 | | 2.685 | | **0.048*** | |
| MXSP - Dual Task (PP) | | | | 3 | | 209.275 | | 2.263 | | 0.082 | |
| SDLP - Single Task (PP) | | | | 3 | | 211.557 | | 15.783 | | **<0.001*** | |
| SDLP - Dual Task (PP) | | | | 3 | | 203.414 | | 2.785 | | **0.042*** | |
| SDSP - Single Task (PP) | | | | 3 | | 224.634 | | 4.030 | | **0.008*** | |
| SDSP - Dual Task (PP) | | | | 3 | | 211.584 | | 3.960 | | **0.009*** | |
| Reaction Time (PP) | | | | 3 | | 194.377 | | 8.039 | | **<0.001*** | |
| ANOVA results assessing the main effect of Δ9-THC potency on driving performance under single and dual task driving conditions. ‘ITT’ (intent-to-treat) analysis includes all participants with at least one drug exposure visit completed, while ‘PP’ (per protocol) analysis is restricted to the n=35 participants who completed the study per protocol (note that PP analysis is reported in the main text). 'MSP' and 'MXSP' refer to mean speed and maximum speed, respectively, 'SDLP' and 'SDSP' denote standard deviation of lateral position and speed. Significant results (*p<0.05) suggest a potency-related effect on driving outcomes. 'NumDF' and 'DenDF' represent numerator and denominator degrees of freedom for the F-test. | | | | | | | | | | | |
| **Table S8.** Pairwise comparisons of Δ9-THC potency effects on driving performance measures (using PP models) | | | | | | | | | | | |
| **Driving Outcome / Driving Condition** | **Contrast** | **Estimate (Δmean**) | | **SE** | | **df** | | **t.ratio** | | **p.value** | |
| MSP - Single Task | High - Low | 0.32 | | 0.53 | | 223.53 | | 0.61 | | 0.54 | |
| MSP - Single Task | High - Med | 0.46 | | 0.54 | | 220.54 | | 0.85 | | 0.40 | |
| MSP - Single Task | High - Pla | 0.89 | | 0.53 | | 221.95 | | 1.67 | | 0.10 | |
| MSP - Single Task | Low - Med | 0.13 | | 0.53 | | 225.28 | | 0.25 | | 0.81 | |
| MSP - Single Task | Low - Pla | 0.56 | | 0.52 | | 221.43 | | 1.07 | | 0.28 | |
| MSP - Single Task | Med - Pla | 0.43 | | 0.53 | | 223.10 | | 0.81 | | 0.42 | |
| MSP - Dual Task | High - Low | 0.70 | | 0.58 | | 223.98 | | 1.20 | | 0.23 | |
| MSP - Dual Task | High - Med | 0.31 | | 0.59 | | 220.68 | | 0.52 | | 0.60 | |
| MSP - Dual Task | High - Pla | 0.83 | | 0.58 | | 222.92 | | 1.41 | | 0.16 | |
| MSP - Dual Task | Low - Med | -0.39 | | 0.58 | | 225.10 | | -0.66 | | 0.51 | |
| MSP - Dual Task | Low - Pla | 0.13 | | 0.57 | | 221.24 | | 0.23 | | 0.82 | |
| MSP - Dual Task | Med - Pla | 0.52 | | 0.59 | | 223.85 | | 0.88 | | 0.38 | |
| MXSP - Single Task | High - Low | 0.87 | | 0.97 | | 223.16 | | 0.90 | | 0.37 | |
| MXSP - Single Task | High - Med | -0.11 | | 0.98 | | 220.09 | | -0.12 | | 0.91 | |
| **MXSP - Single Task** | **High - Pla** | **2.33** | | **0.97** | | **222.13** | | **2.40** | | **0.02** | |
| MXSP - Single Task | Low - Med | -0.98 | | 0.97 | | 224.62 | | -1.01 | | 0.31 | |
| MXSP - Single Task | Low - Pla | 1.46 | | 0.95 | | 220.72 | | 1.53 | | 0.13 | |
| **MXSP - Single Task** | **Med - Pla** | **2.44** | | **0.98** | | **223.70** | | **2.50** | | **0.01** | |
| MXSP - Dual Task | High - Low | 1.15 | | 0.98 | | 223.11 | | 1.18 | | 0.24 | |
| MXSP - Dual Task | High - Med | 1.04 | | 1.00 | | 220.83 | | 1.05 | | 0.30 | |
| **MXSP - Dual Task** | **High - Pla** | **2.55** | | **0.99** | | **221.83** | | **2.59** | | **0.01** | |
| MXSP - Dual Task | Low - Med | -0.11 | | 0.99 | | 224.86 | | -0.11 | | 0.91 | |
| MXSP - Dual Task | Low - Pla | 1.40 | | 0.97 | | 220.42 | | 1.45 | | 0.15 | |
| MXSP - Dual Task | Med - Pla | 1.51 | | 1.00 | | 224.49 | | 1.51 | | 0.13 | |
| **SDLP - Single Task** | **High - Low** | **0.014** | | **0.01** | | **222.08** | | **2.21** | | **0.03** | |
| **SDLP - Single Task** | **High - Med** | **0.013** | | **0.01** | | **219.28** | | **2.11** | | **0.04** | |
| **SDLP - Single Task** | **High - Pla** | **0.041** | | **0.01** | | **221.84** | | **6.66** | | **<0.001** | |
| SDLP - Single Task | Low - Med | 0.001 | | 0.01 | | 223.33 | | -0.07 | | 0.94 | |
| **SDLP - Single Task** | **Low - Pla** | **0.028** | | **0.01** | | **220.13** | | **4.54** | | **<0.001** | |
| **SDLP - Single Task** | **Med - Pla** | **0.029** | | **0.01** | | **223.99** | | **4.49** | | **<0.001** | |
| SDLP - Dual Task | High - Low | 0.005 | | 0.01 | | 223.62 | | 0.45 | | 0.65 | |
| SDLP - Dual Task | High - Med | 0.006 | | 0.01 | | 220.79 | | -0.33 | | 0.74 | |
| **SDLP - Dual Task** | **High - Pla** | **0.025** | | **0.01** | | **222.48** | | **2.31** | | **0.02** | |
| SDLP - Dual Task | Low - Med | -0.011 | | 0.01 | | 225.61 | | -0.77 | | 0.44 | |
| SDLP - Dual Task | Low - Pla | 0.021 | | 0.01 | | 222.05 | | 1.89 | | 0.06 | |
| **SDLP - Dual Task** | **Med - Pla** | **0.032** | | **0.01** | | **223.05** | | **2.63** | | **0.01** | |
| **SDSP - Single Task** | **High - Low** | **0.78** | | **0.35** | | **223.75** | | **2.23** | | **0.03** | |
| **SDSP - Single Task** | **High - Med** | **0.70** | | **0.36** | | **222.77** | | **1.97** | | **0.05** | |
| **SDSP - Single Task** | **High - Pla** | **1.21** | | **0.35** | | **222.15** | | **3.43** | | **<0.001** | |
| SDSP - Single Task | Low - Med | -0.08 | | 0.35 | | 225.35 | | -0.22 | | 0.82 | |
| SDSP - Single Task | Low - Pla | 0.43 | | 0.35 | | 221.89 | | 1.24 | | 0.22 | |
| SDSP - Single Task | Med - Pla | 0.51 | | 0.35 | | 224.10 | | 1.43 | | 0.15 | |
| SDSP - Dual Task | High - Low | 0.58 | | 0.38 | | 225.07 | | 1.52 | | 0.13 | |
| SDSP - Dual Task | High - Med | 0.07 | | 0.39 | | 223.01 | | 0.18 | | 0.85 | |
| **SDSP - Dual Task** | **High - Pla** | **1.16** | | **0.38** | | **224.71** | | **3.03** | | **<0.001** | |
| SDSP - Dual Task | Low - Med | -0.51 | | 0.38 | | 225.12 | | -1.33 | | 0.18 | |
| SDSP - Dual Task | Low - Pla | 0.58 | | 0.37 | | 221.90 | | 1.56 | | 0.12 | |
| **SDSP - Dual Task** | **Med - Pla** | **1.09** | | **0.38** | | **223.66** | | **2.85** | | **<0.001** | |
| **Reaction Time** | **High - Low** | **0.037** | | **0.01** | | **225.77** | | **3.31** | | **<0.001** | |
| Reaction Time | High - Med | 0.007 | | 0.01 | | 222.17 | | 0.43 | | 0.67 | |
| **Reaction Time** | **High - Pla** | **0.048** | | **0.01** | | **224.42** | | **3.98** | | **<0.001** | |
| **Reaction Time** | **Low - Med** | **-0.030** | | **0.01** | | **225.46** | | **-2.88** | | **<0.001** | |
| Reaction Time | Low - Pla | 0.011 | | 0.01 | | 221.77 | | 0.71 | | 0.48 | |
| **Reaction Time** | **Med - Pla** | **0.041** | | **0.01** | | **223.60** | | **3.57** | | **<0.001** | |
| Pairwise comparisons across different measures of driving performance (MSP, MXSP, SDLP, SDSP, RT) under single and dual task driving conditions, comparing placebo (Pla) with low, medium, and high Δ9-THC potencies. Estimates indicate mean differences between conditions, with 'SE' for standard error, and 'df' for degrees of freedom. Negative estimate values suggest an increase relative to the comparison group. Bolded rows (p<0.05) denote statistically significant differences. | | | | | | | | | | | |


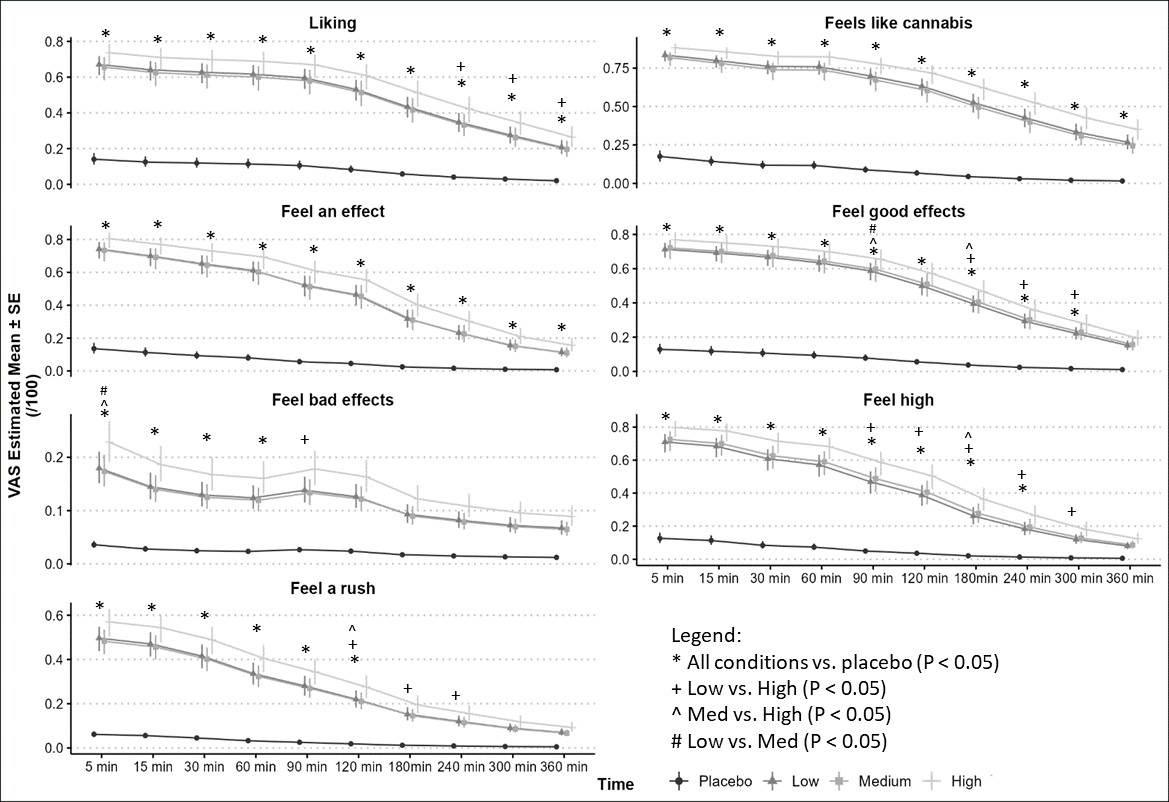
**Figure S3.** Visual Analogue Scales (VAS). Model estimated means ± Standard Error (SE)


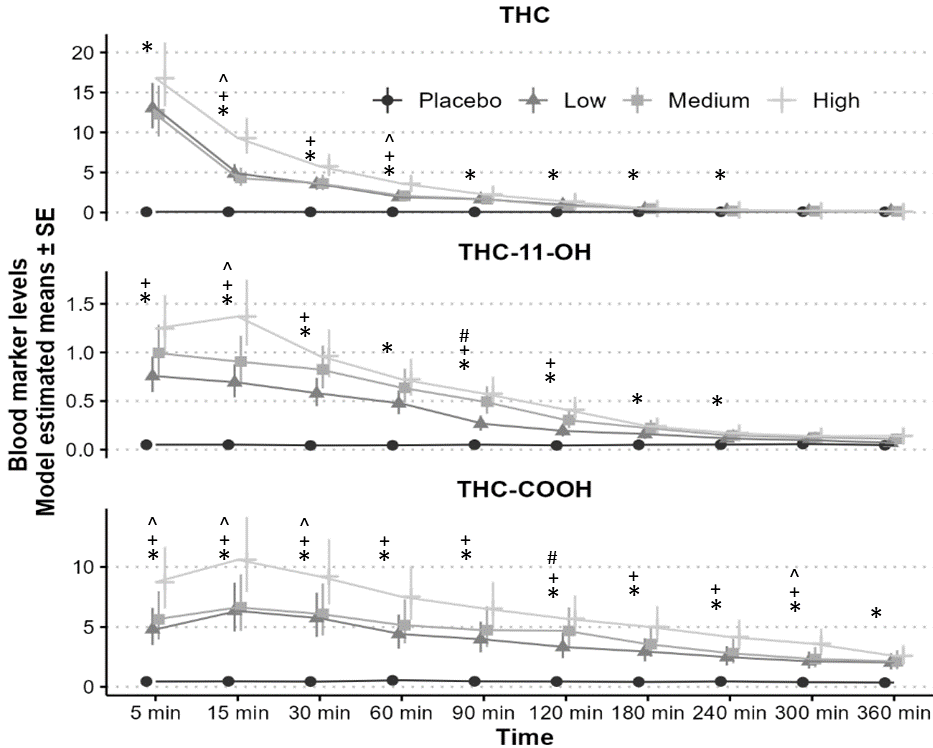


**Figure S4:** Estimated mean Δ9-THC, 11-OH-Δ9-THC, and Δ9-THC-COOH concentrations in blood over time (ng/ml ).

|  | **Table S9** Estimated slope for the blood marker for the model not controlling (or controlling*) for condition and including baseline | | | | | | | |
| --- | --- | --- | --- | --- | --- | --- | --- | --- |
| **Substance** | **Measure** | **Slope** | **SE** | **t.ratio** | **p value** | **p value*** | **r** | **Confidence Interval** |
| Δ9-THC | Reaction Time | 0.002 | 0.0009 | 2.28 | **0.023** | 0.090 | 0.120 | (0.016, 0.220) |
| Δ9-THC | SDLP | 0.0024 | 0.0006 | 3.94 | **<0.001** | 0.050 | 0.206 | (0.104, 0.301) |
| Δ9-THC | After | -0.086 | 0.014 | -5.93 | **<0.001** | 0.488 | -0.371 | (-0.473, -0.254) |
| Δ9-THC | Skill | -0.038 | 0.013 | -2.89 | **0.004** | **0.030** | -0.152 | (-0.250, -0.049) |
| Δ9-THC | Willing | -0.092 | 0.018 | -4.96 | **<0.001** | **<0.001** | -0.255 | (-0.347, -0.156) |
| 11-OH-Δ9-THC | Reaction Time | 0.0069 | 0.0032 | 2.15 | **0.031** | 0.116 | 0.114 | (0.010, 0.214) |
| 11-OH-Δ9-THC | SDLP | 0.0057 | 0.0019 | 2.96 | **0.003** | 0.137 | 0.156 | (0.052, 0.254) |
| 11-OH-Δ9-THC | After | -0.321 | 0.050 | -6.47 | **<0.001** | 0.071 | -0.400 | (-0.498, -0.285) |
| 11-OH-Δ9-THC | Skill | -0.147 | 0.042 | -3.47 | **<0.001** | **0.006** | -0.182 | (-0.278, -0.079) |
| 11-OH-Δ9-THC | Willing | -0.268 | 0.058 | -4.60 | **<0.001** | **<0.001** | -0.238 | (-0.331, -0.138) |
| Δ9-THC-COOH | Reaction Time | 0.00056 | 0.0004 | 1.57 | 0.116 | **0.034** | 0.083 | (-0.021, 0.185) |
| Δ9-THC-COOH | SDLP | 0.0012 | 0.0002 | 4.86 | **<0.001** | **0.003** | 0.251 | (0.151, 0.343) |
| Δ9-THC-COOH | After | -0.049 | 0.0072 | -6.77 | **<0.001** | 0.101 | -0.415 | (-0.511, -0.302) |
| Δ9-THC-COOH | Skill | -0.020 | 0.0048 | -4.31 | **<0.001** | **0.001** | -0.224 | (-0.318, -0.123) |
| Δ9-THC-COOH | Willing | -0.022 | 0.0055 | -3.95 | **<0.001** | **<0.001** | -0.206 | (-0.301, -0.104) |
|  | Δ9-THC (Δ9-Tetrahydrocannabinol) 11-OH-Δ9-THC (11-Hydroxy-Δ9-tetrahydrocannabinol) Δ9-THC-COOH (11-Nor-9-carboxy-Δ9-tetrahydrocannabinol)  *Adjusted model controlling for condition | | | | | | | |

**Figure S5.** Vital Signs. Model estimated means ± Standard Error (SE).**
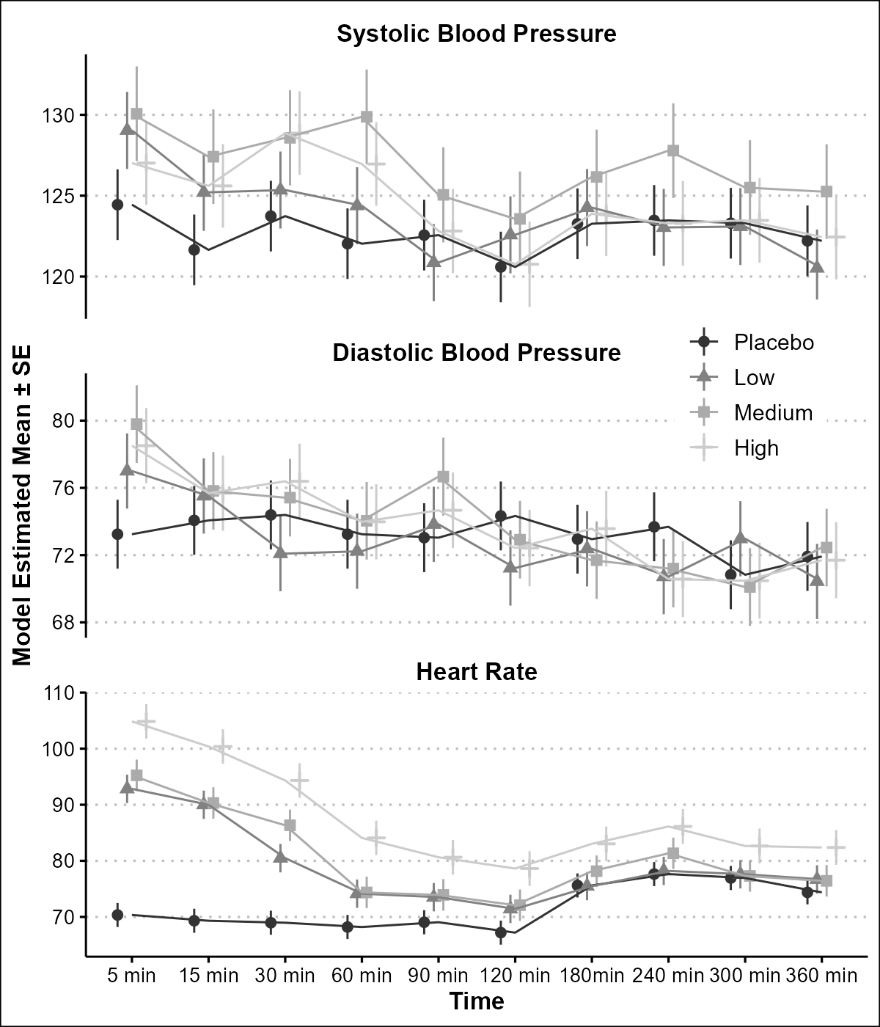
**
Blood pressure (mmHg). Heart rate (beats/minute)**
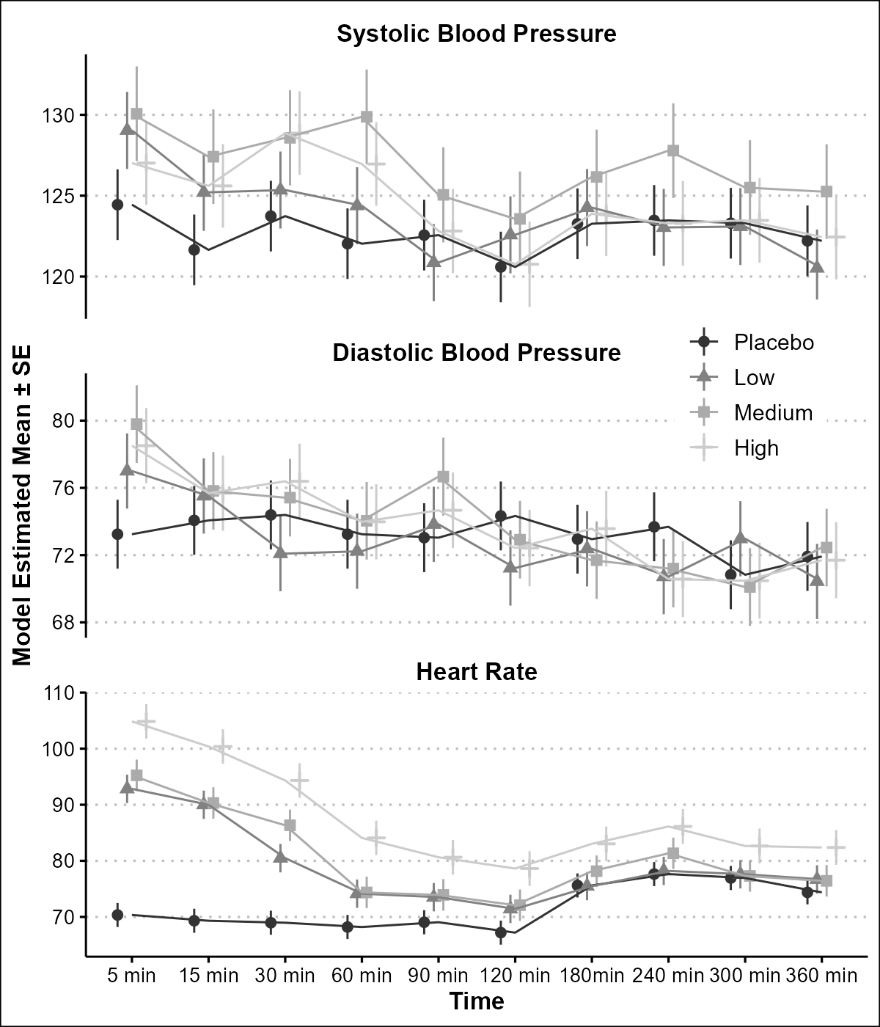
**

1. Sobell LC, Sobell MB. Timeline follow-back: A technique for assessing self-reported alcohol consumption. Measuring alcohol consumption: Psychosocial and biochemical methods. Totowa, NJ, US: Humana Press/Springer Nature; 1992:41-72.

2. Fares A, Wickens CM, Mann RE, et al. Combined effect of alcohol and cannabis on simulated driving. Psychopharmacology (Berl) 2022;239(5):1263-1277. (In eng). DOI: 10.1007/s00213-021-05773-3.

3. Green P, MacLeod CJ. SIMR: an R package for power analysis of generalized linear mixed models by simulation. Methods in Ecology and Evolution 2016;7(4):493-498. DOI: <https://doi.org/10.1111/2041-210X.12504>.
